# Relation between Urine Cytological Findings and Renal Function in Patients with Kidney Stones in Taif, Saudi Arabia

**DOI:** 10.3390/medicina60101630

**Published:** 2024-10-05

**Authors:** Sahar Ali Qahtani, Khadiga A. Ismail, Howaida M. Hagag, Maram Jamel Hulbah, Maha M. Bakhuraysah, Nidaa Mahmoud Johari, Salman Mohammed Alotaibi, Seham Alajmani, Hani Diafallah Alseyali, Manal Ali Ayoub, Khalid Abdullah Althagafi, Ali Awad Alnofaie, Abdulbadea Dawod Abdulaziz, Abdulhadi Samman, Hussain Noorwali, Mohammed S. Abdelwahed, Abdulkarim Hasan

**Affiliations:** 1 Department of Clinical Laboratory Sciences, Poison Control and Forensic Chemistry Center, Jeddah 21176, Saudi Arabia; 2 Department of Clinical Laboratory Sciences, College of Applied Medical Sciences, Taif University, Taif 21944, Saudi Arabia; 3 Department of Clinical Laboratory Sciences, Aljawdah Laboratory Company, Taif 72701, Saudi Arabia; 4 Department of Clinical Laboratory Sciences, Ministry of Defense—Saudi Armed Forces, Riyadh 11159, Saudi Arabia; 5 Department of Laboratory, King Faisal Medical Complex, Taif 21944, Saudi Arabia; 6 Department of Training and Learning, King Faisal Medical Complex, Taif 21944, Saudi Arabia; 7 Department of Basic Medical Sciences, Pathology Division, College of Medicine, University of Jeddah, Jeddah 21589, Saudi Arabia; 8 Department of Pathology, Faculty of Medicine, Al-Azhar University, Cairo 11884, Egypt; 9 Department of Laboratory, Prince Mishari bin Saud Hospital, Al-Baha Health Cluster, A-Baha 65784, Saudi Arabia

**Keywords:** kidney stones, papanicolaou stain, eGFR, nuclear atypia, urine cytology

## Abstract

*Background and Objectives*: Urine serves as a vital diagnostic fluid, and urine cytology analysis plays a crucial role in identifying urinary system illnesses such as bladder cancer and kidney stones. The Paris System for Reporting Urinary Cytology establishes a uniform method for diagnosing urinary tract cancer. This study aimed to provide valuable insights that can inform diagnostic strategies related to kidney stones and ultimately improve patient outcomes via the early detection of the cellular changes associated with kidney stones and their relation to kidney function tests. *Materials and Methods*: A comparative study was conducted and comprised two groups: group 1, consisting of 50 patients diagnosed with kidney stones, and group 2, comprising 50 patients diagnosed with other kidney diseases. Renal function tests and urinalysis (via the PAP staining of urine cellular deposits to detect nuclear changes) were performed, and the results were analyzed. *Results*: There was a statistically significant increase in urinary red blood cells, white blood cells, and nuclear reactive atypical changes in urinary sediments of kidney stone patients compared to the patients without stones, while there was a decrease in the estimated glomerular filtration rate (eGFR). eGFR showed a 96.7% specificity in detecting cases with nuclear reactive atypia. *Conclusions*: eGFR emerges as a reliable diagnostic marker for the comprehensive assessment of kidney stones, particularly when associated with nuclear atypia. The significant correlation between the indicators of chronic kidney disease, such as decreased eGFR, and the presence of kidney stones emphasizes the urgent need for efficient diagnostic practices.

## 1. Introduction

Kidney stones are solid masses formed from crystal aggregations in one or both kidneys, varying in size from millimeters to centimeters. While most stones pass through the urinary tract with ease, some require medical intervention for their removal. These stones can consist of phosphate, uric acid, magnesium ammonium phosphate, apatite, or struvite flakes. Notably, around 75% of urinary calculi are calcium-containing stones, comprising pure calcium crystals (50%), calcium–phosphate crystals (5%), or a combination of both (45%) [1].

The genesis of kidney stones is primarily attributed to two factors: injury to renal tubular epithelial cells and salt supersaturation in urine. High oxalate levels can damage both proximal and distal kidney tubular cells. Ferric oxide and autophagy play significant roles in kidney injury, with hyperoxaluria and calcium oxalate crystals further affecting tubular epithelial cells, leading to excessive autophagy stimulation, cell damage, and death. Ferroptosis, an iron-dependent cell death process characterized by lipid peroxide accumulation and reduced glutathione peroxidase 4 (GPX4) activity, also contributes to this process [2]. The annual incidence of urolithiasis in the Western world is approximately 0.5%, with a lifetime risk ranging from 10% to 15%; however, the incidence in the Middle East is even higher, by 20% to 25% [1].

Urine, one of the most frequently analyzed cytologic collections and often referred to as the liquid gold of diagnostics serves as a convenient screening tool with which physicians can assess patients with urinary symptoms. It also aids researchers in monitoring patients with previous bladder cancer [3,4].

Urine cytology is a cost-effective and safe method for detecting anomalies in the urinary system and identifying various disorders, including bacterial infections and recurrent urothelial cancer. However, the sensitivity and specificity of urine cytology may vary depending on the sampling method and tumor grade [5].

Cytologists primarily focus on diagnosing premalignant and malignant disorders, with cytology serving as a crucial cancer-screening method. The accurate diagnosis of infectious diseases through cytodiagnosis enables timely and appropriate medical care. While infections are easier to identify in cytological preparations compared to structural specimens, histopathological examination remains highly effective in detecting host responses to pathogens. Giemsa staining, in addition to standard PAP staining, is recommended when viral infections are suspected [6].

Urine sediment analysis is an integral part of urine cytology investigations, aiding in the differentiation of hematuria from the upper and/or lower urinary tracts and the diagnosis of various benign conditions. Indications for urine cytological verification include the detection of non-neoplastic, premalignant, and malignant lesions, the screening of asymptomatic patients exposed to carcinogens, and monitoring therapy. However, the cytological diagnosis of low-grade tumors is challenging due to the minimal morphological differences between normal cells and the exfoliated cellular groups of low-grade tumors [7].

Following the International Congress of Cytology in Paris, the Paris System for Reporting Urinary Cytology (TPS) was established as the first standardized, evidence-based reporting system for urine specimens. Its primary objective is to reduce unnecessary ambiguous diagnoses while preserving urine cytology’s superior ability to identify high-grade urothelial cancer [8].

Urine samples with bacteriuria, whether confirmed or suspected, can be broadly classified into rods versus cocci using Gram staining. Monolayer liquid-based cytology techniques and PAP staining are commonly employed due to the significant role of nuclear morphology in cytology. PAP staining enhances the visibility of chromatin and nuclear membranes, which are crucial elements in grading systems for standard human cytopathology. Despite its benefits, the Papanicolaou staining of cytology specimens has not gained widespread popularity in medicine [9].

Chronic kidney disease (CKD) results from a permanent alteration in kidney structure or function, characterized by gradual and irreversible degeneration. CKD pathology indicates a higher risk of complications, particularly those related to the cardiovascular system [10].

Recent research by Palaoro et al. [11] highlighted the presence of sediments in urine samples, which were evident in both fresh specimens and smears stained using the PAP method. This underscores the critical importance of conducting cytological examinations on fresh urine samples and subsequently confirming diagnoses with PAP staining. Such comprehensive diagnostic procedures serve to not only diagnose tumoral or nontumoral pathologies but also screen for precancerous lesions or carcinoma in situ.

Building upon this foundation, we seek to understand the impact of kidney stones and their associated cellular changes on renal function. Additionally, we endeavor to identify the markers that indicate an affected kidney function. By investigating these cellular phenomena within the context of the prevalent kidney diseases in our region, we aim to provide valuable insights that can inform diagnostic strategies for managing complications related to kidney stones and ultimately improve patient outcomes via the early detection of cellular changes associated with kidney stones and their relation to kidney function tests in comparison to other non-neoplastic kidney diseases.

## 2. Materials and Methods

### 2.1. Patients and Settings

A comparative study involving two groups was conducted at the King Faisal Medical Complex (KFMC): group 1 consisted of 50 patients presenting with kidney stones; group 2 included 50 patients diagnosed with other kidney diseases without kidney stones including CKD (early-stage only). The study was carried out at the College of Applied Medical Sciences, Clinical Laboratory Sciences Department, with patient samples and data obtained from the parasitology department at KFMC in Taif between August 2023 and May 2024 and diagnosed via on-site and remote cytological examination. Patients aged 30 to 70 years were included in the study. This age group was chosen for its likelihood of active participation and a higher incidence of the events under investigation. Additionally, participants were required to have resided in Taif for at least nine months to ensure acclimatization to the local environment. The exclusion criteria included an inability or unwillingness to participate and the presence of morbid diseases such as cancer and late-stage CKD. To achieve an 80% power to reject the null hypothesis, the sample size was calculated according to the following equation:

*N* = z2 × P(1-P)/ɛ2

*N* = (1.04)2 × 0.7(1-0.7)/(0.07)^2^~50

### 2.2. Laboratory Investigations

Blood samples were taken for the analysis of glucose, urea, creatinine, and uric acid using a Cobas e 601 device (BIO-MED, serial number 28H3-13, Roche Molecular Systems, Inc., Pleasanton, CA, USA). Additionally, a blood count was conducted using a hematology analyzer (Sysmex 9000, serial number 32117, Europe SE).

Urine samples were collected, though first-pass urine samples were rejected due to abnormal cell features, such as enlarged nuclei and washed-out chromatin, resembling malignant cells. Subsequent urine analyses were performed using the Lab UMat 2 and Urised 3 Pro devices (77 Elektronika, serial number UPA 05000452, Hungary (EU), and the results were recorded. Urine cellular sediments were stained with a special PAP stain to detect DNA damage.

The Paris System for Reporting Urinary Cytology (TPS 1.0, published in 2016) represents the first standardized, evidence-based reporting system established as an international standard for urine specimen reporting post the International Congress of Cytology in Paris. Its primary objective is to decrease the rate of unnecessary indeterminate diagnoses while maintaining the excellent performance of urine cytology in detecting high-grade urothelial carcinoma. The reporting system consists of the following diagnostic categories: negative for high-grade urothelial carcinoma (NHGUC), atypical urothelial cells (AUCs), and suspicious for high-grade urothelial carcinoma (SHGUC) and high-grade urothelial carcinoma (HGUC) (Chen and Lin, 2023) [8]. We use the term of nuclear atypia in case of the presence of reactive nuclear changes.

The estimated glomerular filtration rate is calculated from the following equation:

eGFRcr = 142 × min(Scr/κ, 1)α × max(Scr/κ, 1) − 1.200 × 0.9938Age × 1.012 [mL/min/1.73 m^2^]

where Scr = standardized serum creatinine in mg/dL; κ = 0.7 (female) or 0.9 (male); α = −0.241 (female) or −0.302 (male); min(Scr/κ, 1) is the minimum Scr/κ or 1.0; max(Scr/κ, 1) is the maximum Scr/κ or 1.0.

The cut-off value is 60 mL/min/1.73 m^2^ below the considered kidney impairment according to Qu et al. [12].

### 2.3. Data Processing and Statistical Analysis

The results were analyzed using the computerized software program SPSS version 23. A Chi-square test, exact Fisher test, and *t*-test were employed for statistical analysis. A probability value below 0.05 was deemed statistically significant.

## 3. Ethical Considerations

The study received ethical approval from the Ethics Committee of Taif Health Cluster, KFMC (H-02-T-123/2024-E-30).

## 4. Results

Our study included 100 patients, with 50 cases presenting with kidney stones and 50 diagnosed with kidney diseases other than stones (CKD). The mean age and standard deviation in group 1 were 50.1 ± 12.1, and, in group 2, they were 46.5 ± 15.6, with no statistically significant difference observed (*p* = 0.20).

**Figure 1:**
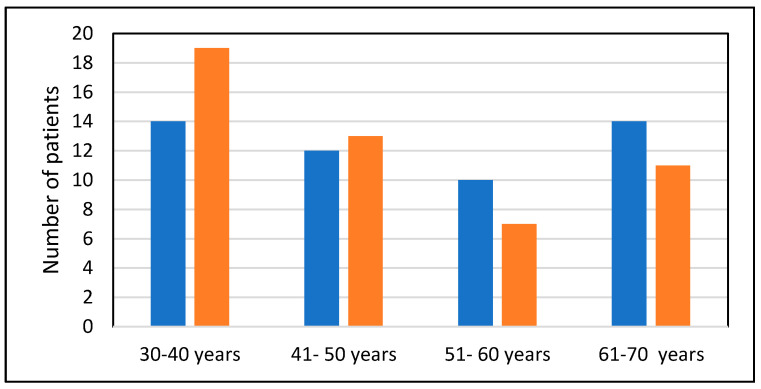
Age groups’ distribution among the two groups (blue column = group 1 and red column = group 2).

Age group distribution analysis revealed that the most common age groups among cases were from 30 to 40 years and 61 to 70 years (Figure 1).

Regarding sex distribution, 48% (24/50) of group 1 were males, and 52% (26/50) were females, while 34% (17/50) of group 2 were males, and 66% (33/50) were females, with no statistically significant difference noted (*p* = 0.155).

A microscopic examination of urine samples demonstrated a significant increase in the detection of urinary RBCs, WBCs, and nuclear atypia in group 1 compared to group 2 (*p* < 0.01). Hematological and chemical parameters analysis showed a significant elevation in urea (*p* = 0.04), and creatinine (*p* = 0.01), and a decrease in eGFR (*p* = 0.04) in cases of CKD compared to cases of stones (Table 1).

Table 2 shows a less significant increase in the level of urea, creatinine, and eGFR in group 1 than in group 2. Non-statistically significant is age, WBC, glucose, and uric acid in both groups.

A significant association between creatinine and eGFR in cases with nuclear atypia has been detected (Table 3).

In Table 4, the eGFR has high specificity in detection cases with nuclear atypia.

**Figure 2:**
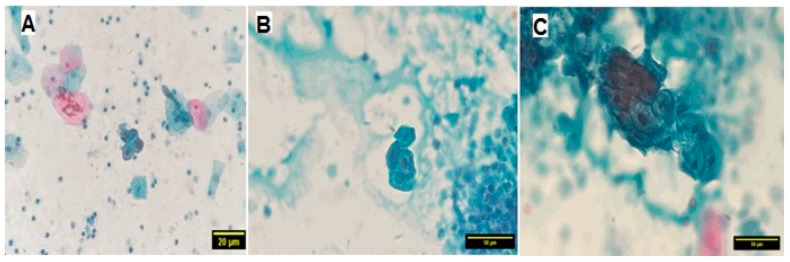
(**A**): PAP stain 20×—urine sample. The cells show squamous cells from urethral contamination and reactive urothelial cells with nuclear atypia, deep-stained cells, and high nuclear:cytoplasmic ratio. The background contains inflammation cells. (**B**): PAP stain 40×—urine sample. The cells show reactive urothelial cells with nuclear hyperchromasia (atypia), deep-stained cells, and high nuclear:cytoplasmic ratio. (**C**): PAP stain 40×—urine sample. The background contains inflammation cells. The cells show reactive urothelial cells with nuclear atypia, high nuclear:cytoplasmic ratio, and irregular nuclear membrane.

Figure 2 displayed light microscope images of urine samples stained by PAP stain, illustrating nuclear atypia in cells.

## 5. Discussion

Kidney stone disease, or nephrolithiasis, occurs when the urinary solutes precipitate within the urinary space to form aggregates of crystalline material. The incidence has been increasing, and demographics have been evolving [13,14]. Once viewed as a simple disease with intermittent exacerbations simply managed by urologists, kidney stone disease is now recognized as a complex medical and surgical condition requiring careful evaluation and multifaceted care [14,15].

The occurrence of kidney stone disease poses a significant global health concern, impacting individuals across diverse demographics and geographical regions. In Saudi Arabia, where cultural and dietary practices are unique, the prevalence of kidney stones has garnered increasing attention within public health. As the Kingdom experiences rapid socioeconomic development and urbanization, accompanied by shifts in lifestyle and dietary habits, comprehending the epidemiology and factors contributing to kidney stone formation becomes imperative for healthcare professionals and policymakers [15]. Few previous studies have tried to estimate how prevalent kidney stones are locally in the country, with various percentages being reported. In 2015, Ahmad et al. reported 14% nephrolithiasis frequency among patients who have had it be managed at a local clinic in Riyadh [16]. However, Safdar et al. [17], in 2021, concluded that the prevalence of kidney stones was nearly 9%.

Studies have highlighted an association between kidney stone disease and chronic kidney function impairment. However, not all stone formers develop chronic kidney function impairment, and various factors contribute to renal function damage in these patients. These factors encompass anatomical abnormalities, infections, underlying metabolic disorders, environmental influences, repeated interventions, dietary factors, and molecular or genetic factors. Hypertension and diabetes, established risk factors, have been significantly associated with chronic kidney functional impairment in both the general population and stone formers [18].

When interpreting our findings in comparison with existing studies, it is essential to acknowledge potential variability attributable to several factors. Demographic differences, such as geographic location and ethnicity, significantly influence the age distribution of kidney stone patients and those with other kidney diseases. Variations in sample size and study design across different research endeavors may also impact the observed age patterns. Moreover, discrepancies in defining and diagnosing kidney stone patients versus those with other kidney diseases could contribute to variations in the age distribution within each group. Notably, our study found no significant relation between age groups and renal stone incidence.

Similarly, the comparison of sex distribution findings with other studies requires a nuanced understanding of potential influencing factors. Variations in healthcare-seeking behavior and societal influences may significantly affect the prevalence rates of kidney stone cases and other kidney diseases among males and females. For instance, a higher prevalence of kidney stones was observed in specific demographic subgroups in other studies, emphasizing the complex interplay of factors influencing disease prevalence [19].

This study aimed to elucidate the pathological processes associated with kidney stones by examining various biochemical and cytological parameters. Our findings were compared with the existing literature to provide insights into the impact and progression of kidney stones. Consistent with prior research, our study observed a significant presence of RBCs in the urine (hematuria) of 24% of kidney stone patients. This aligns with the established understanding that stones moving within the urinary tract can cause physical trauma, leading to hematuria [20]. Additionally, our findings corroborate previous research indicating a 35% positivity rate for erythrocytes in urine analysis [21].

Our study also found a statistically significant presence of white blood cells (WBCs) in the urine samples of 44% of kidney stone patients. This suggests a potential co-occurring infection or inflammation, commonly seen in patients with obstructive urolithiasis [22]. In such cases, urinary stones can obstruct urine flow, leading to bacterial growth and inflammatory responses [23]. These findings contrast with another study, which reported a significant WBC presence in only 10% of female kidney stone patients [19].

The measured biochemical markers in our study, including urea (29.3 ± 18.2 mg/dL), creatinine (1.0 ± 0.4 mg/dL), and eGFR at 87.8 ± 26.6 mL/min/1.73 m^2^, provide valuable insights into kidney function. Serum creatinine, a late marker of acute kidney injury, typically rises only after the significant loss of kidney function [24]. Our observed urea and creatinine levels suggest a relatively normal renal function in most cases of kidney stones, despite statistically significant elevations compared to cases without kidney stones. This contrasts with studies reporting higher creatinine levels and lower eGFRs, indicating a more severe kidney impairment associated with kidney stones. Additionally, our findings suggest a comparable risk of a sustained eGFR below 60 mL/min per 1.73 m^2^ in individuals with kidney stones, although statistically significant, contrasting with the previous research conducted by Moludi et al. [25], Norris et al. [26], and Rule et al. [27].

Cellular analysis revealed nuclear changes in 16% (8/50) of kidney stone patients, characterized by nuclear enlargement, hyperchromasia, or irregular contours. These changes may signify cellular stress or damage associated with the inflammatory or obstructive nature of kidney stones. The absence of such changes in 84% of patients suggests that these alterations may be more prevalent in individuals with advanced or symptomatic stone disease. Previous studies have demonstrated a correlation between nuclear changes and the severity of obstruction or presence of infection, supporting our findings (Alelign and Petros [1]; Rule et al. [27]).

Interestingly, our analysis revealed that, among patients specifically categorized under kidney inflammation without stone pathology, no nuclear changes were observed in any case. This finding suggests that the inflammatory processes in these patients may be less likely to cause direct cellular damage or that the inflammation manifests as a milder form not leading to detectable cytological alterations [28]. This observation underscores potential differences in the pathophysiology of kidney stones compared to other forms of kidney inflammation, such as glomerulonephritis or interstitial nephritis, where nuclear changes may be more pronounced due to distinct underlying mechanisms [29,30].

Furthermore, exploring the significance of eGFR in kidney stone patients raises questions about its relationship with nuclear atypia and its diagnostic utility. While eGFR serves as a widely accepted indicator of kidney function, its correlation with specific histopathological features such as nuclear reactive atypia in kidney stone patients remains variable. Nonetheless, the significant difference in eGFR between both groups (*p* < 0.001) aligns with the study of Sigurjonsdottir et al. [31], which reported a higher prevalence of CKD among stone formers compared to controls (*p* < 0.001). Additionally, hypertension and diabetes were significantly more prevalent among cases with higher BMIs compared to the other group. Stone formers with a lower eGFR exhibited an elevated supersaturation in uric acid but a reduced supersaturation in calcium-containing stones. Generally, the kidney stones can consist of phosphate, uric acid, magnesium ammonium phosphate, apatite, or struvite flakes, and such stones can be impacted on lining cells of the urinary tract causing destruction allowing blood to leak into urine [32,33]. One of this study’s limitations is the inability to assess the type of stone and to study the correlations. Furthermore, since this study was held in one region, Taif, it has been difficult to study people from other regions in the Kingdom, which could have emphasized lifestyle differences and geographical distribution.

## 6. Conclusions

This study underscores the persistent health issue presented by kidney stones, especially in Saudi Arabia given its distinctive cultural and dietary norms. We emphasize the importance of utilizing eGFR in conjunction with nuclear changes (reactive atypia) in urine cytology to provide robust diagnostic indicators for a thorough evaluation of kidney stones. The notable association between markers of chronic kidney disease, such as decreased eGFR, and the presence of kidney stones underscores the necessity of effective diagnostic strategies.

## Figures and Tables

**Table 1:** Sex and urine microscopic examination parameters in both groups.

		Studied Groups			
		Group 1 (n = 50)	Group 2 (n = 50)	X2	*p* Value
Sex	Male (n = 41)	24 (48%)	17 (34%)	2.02	0.155
	Female (n = 59)	26 (52%)	33 (66%)		
Urine RBCs Microscopic detected	0	20 (40%)	44 (88%)	31.2	<0.01
	+1	8 (16%)	6 (12%)		
	+2	8 (16%)	0 (0%)		
	+3	12 (24%)	0 (0%)		
	+4	2 (4%)	0 (0%)		
Urine WBCs Microscopic detected	0	11 (22%)	30 (60%)	27.9	<0.01
	+1	22 (44%)	3 (6%)		
	+2	5 (10%)	8 (16%)		
	+3	8 (16%)	9 (18%)		
	+4	4 (8%)	0 (0%)		
Nuclear atypia by PAP stain	Present	8 (16%)	0 (0%)	8.696	0.003
	Absent	42 (84%)	50 (100%)		

**Table 2:** Age, hematological and chemical parameters in both groups.

	Group 1		Group 2		*p* Value
	Mean	SD	Mean	SD	
Age	46.5	15.6	50.1	12.1	0.20
WBC^#^	6.6	1.9	7.3	3.4	0.21
Glucose	119.5	59.6	109.2	36.4	0.30
Uric acid	5.7	1.3	5.5	1.4	0.53
Urea	23.5	9.0	29.3	18.2	0.04
Creatinine	0.8	0.2	1.0	0.4	0.01
eGFR	87.8	26.6	97.6400	19.75747	0.04

**Table 3:** Difference between nuclear atypia by PAP stain with hematological and chemical examination parameters in kidney stone patients.

		Atypia (n = 8)	No Atypia (n = 42)	*t*	*p* Value
Age (Y)	Mean	57.4	48.7	−1.914	0.062
	SD	12.6	11.6		
WBC (×10^3^)	Mean	5.7	7.6	1.514	0.137
	SD	2.7	3.5		
Glucose	Mean	110.0	109.1	−0.063	0.950
	SD	39.0	36.4		
Uric acid	Mean	5.2	5.6	0.662	0.511
	SD	1.2	1.5		
Urea	Mean	20.9	30.9	1.431	0.159
	SD	17.5	18.1		
Creatinine	Mean	1.3	0.9	−2.528	0.015
	SD	0.5	0.4		
eGFR	Mean	62.0	92.8	3.284	0.002
	SD	24.2	24.3		

**Table 4:** Diagnostic performance of eGFR in patients with kidney stones associated with nuclear atypia in urinary cellular deposits.

		Nuclear Atypia		Sensitivity % (95% CI)	Specificity % (95% CI)	PPV % (95% CI)	NPV % (95% CI)	Agreements % (Kappa Test)
		Positive	Negative					
eGFR	Positive	6	3	75% (34.9% to 96.8%)	96.7% (90.7% to 99.3%)	66.7% (38% to 86.7%)	97.8% (93.1% to 99.3%)	95% (88.7% to 98.3%)
	Negative	2	89					

Abbreviations: CI: confidence intervals; PPV, positive predictive value; NPV, negative predictive value.

## Data Availability

All data supporting the reported results are available upon formal request.
